# Fascinating wonderful network: Rete mirabile of the maxillary artery in cats – minireview

**DOI:** 10.1007/s11259-023-10181-3

**Published:** 2023-07-31

**Authors:** Filip Korim, Mária Kuricová, Katarína Vdoviaková, Lenka Krešáková

**Affiliations:** 1 Department of Morphological Disciplines, University of Veterinary Medicine and Pharmacy in Košice, Komenského 73, Košice, 041 81 Slovak Republic; 2 Small Animal Clinic, University Veterinary Hospital, University of Veterinary Medicine and Pharmacy in Košice, Komenského 73, Košice, 041 81 Slovak Republic

**Keywords:** Arteries, Cat, Corrosion casting, Maxillary artery, Rete mirabile

## Abstract

Cats are one of the most common companion animals, and they differ from dogs in several important ways. Considering the central importance of anatomy in high-quality medicine, the treatment of the feline mandible, mostly during intraoral procedures requiring general anaesthesia, has many important features. In cats, the major artery of the brain is the maxillary artery that forms unique structure – the rete mirabile. The rete mirabile is a plexus like vascular structure that lies extracranially and communicates with brain arterial circle through the orbital fissure. The development of the brain vasculature is different in cats, and it includes obliteration mechanisms of the internal carotid artery. The course of the maxillary artery that forms the rete mirabile has a strong relationship to the angular process of the mandible. Emphasis should be placed on manipulation with the feline mandible, especially during open-mouth procedures, as mistakes can lead to blindness, deafness, and central neurological disorders due to compression of the maxillary artery by the angular process of the mandible. This paper focuses on the anatomy and function of the blood supply to the brain, which is very specific in domestic cats and other felids.

## Introduction

From the perspective of comparative anatomy, the arterial supply to the brain is variable in domestic mammals, and there are many different pathways of blood supply to the brain provided by the branches of the common carotid artery (Table 1) (König and Liebich 2020; Popesko 1992; Singh 2017). The internal carotid artery (ICA) and the external carotid artery (ECA) branch off from the common carotid artery (CCA). These branches are responsible for the vascular supply to the head (extracranial and intracranial structures) (König and Liebich 2020; Popesko 1992; Singh 2017). In carnivores, there are different pathways of arterial blood supply between the *Canidae* family and the *Felidae* family. In dogs, the internal carotid artery is the dominant and major artery of the brain. In felids, the arterial system of the brain is supplied by different routes, and the main artery of the brain is the maxillary artery (MA) (Getty 1975; Gillilan 1976; Kiełtyka-Kurc et al. 2015; Takemura 1982). The maxillary artery forms a specific and unique network in the pterygoid fossa called the rete mirabile, which consists of retial branches. The retial branches pass through the orbital fissure into the cranial cavity where, together with other cerebral arteries, they form the cerebral arterial circle (Kier et al. 2019; Ziemak et al. 2021). The development of the cerebral supply is complicated in cats and involves mechanisms of obliteration of the internal carotid artery (Ziemak et al. 2021). The caudal supply is present and corresponds to that of other animals, being represented by the basilar artery (Popesko 1992). The presence of the rete mirabile of the MA is the major difference in the arterial supply to the brain in cats and even-toed ungulates. In this review article, the vascular supply of the brain in cats is explained and described, with most attention given to the rete mirabile of the maxillary artery. The clinical significance of this structure is also discussed. The article renews information on the cerebral vessels in cats that is generally lacking in available anatomic books. This article has a strong educational significance because of the explanatory figures.

**Table 1:** Comparative anatomy of the arterial brain supply in the domestic mammals (Getty 1975; König and Liebich 2020; Popesko 1992)

Horses	Ruminants	Pigs	Dogs	Cats
cerebral arterial circle	cerebral arterial circle	cerebral arterial circle	cerebral arterial circle	cerebral arterial circle
internal carotid artery	rostral epidural rete mirabile	rostral epidural rete mirabile	internal carotid artery	rete mirabile of the maxillary artery (anastomotic branch)
	caudal epidural rete mirabile	caudal epidural rete mirabile		
basilar artery	basilar artery	internal carotid artery	basilar artery	basilar artery
	internal carotid artery (foetuses)	basilar artery		internal carotid artery (foetuses)

## Material and methods

Based on a search of PubMed, Web of Science, and Google Scholar, 28 scientific articles were selected and evaluated. Keywords were used such as: Cat, carotid artery, dentistry, internal carotid artery, maxillary artery, rete maxillaris and rete mirabile. The information was taken from articles that used anatomical dissection methods such as corrosion casting and latex injection followed by dissection. Imaging contrast studies (CT, MRI and X-ray) were also considered. Special attention was paid to the articles with clinically significant and clinically relevant information. The anatomical specimens used in this review were prepared by the authors themselves. The corrosion casting method was used to represent the extracranial and intracranial arteries, using acrylic resin as the casting agent. The specimens were examined under the Carl Zeiss Movena S7 surgical microscope (Carl Zeiss AG, Germany) to obtain detailed images of the rete mirabile. Five corrosive casts from mix-breed shorthaired cats (*Felis catus*) cadavers (3 young, 2 adults), with an average age of 2 years (8 months to 4 years) were used and randomly chosen for photographing the rete mirabile of the maxillary artery for this review article. The animals were humanly euthanized for fatal traumatic injuries with poor prognosis and not for purposes of this article. For corrosion casting, a self-curing resin was applied through the ascending aorta. After resin polymerisation, cadavers were macerated in 2% sodium hydroxide for 2–4 days. We performed a detailed inspection of the specimen and took pictures of the rete mirabile of the maxillary artery and other arteries of the head of specimens previously prepared, which serve as a part of scientific and educational studies at our institution.

## Internal carotid artery in cat and its fate

**Fig. 1:**
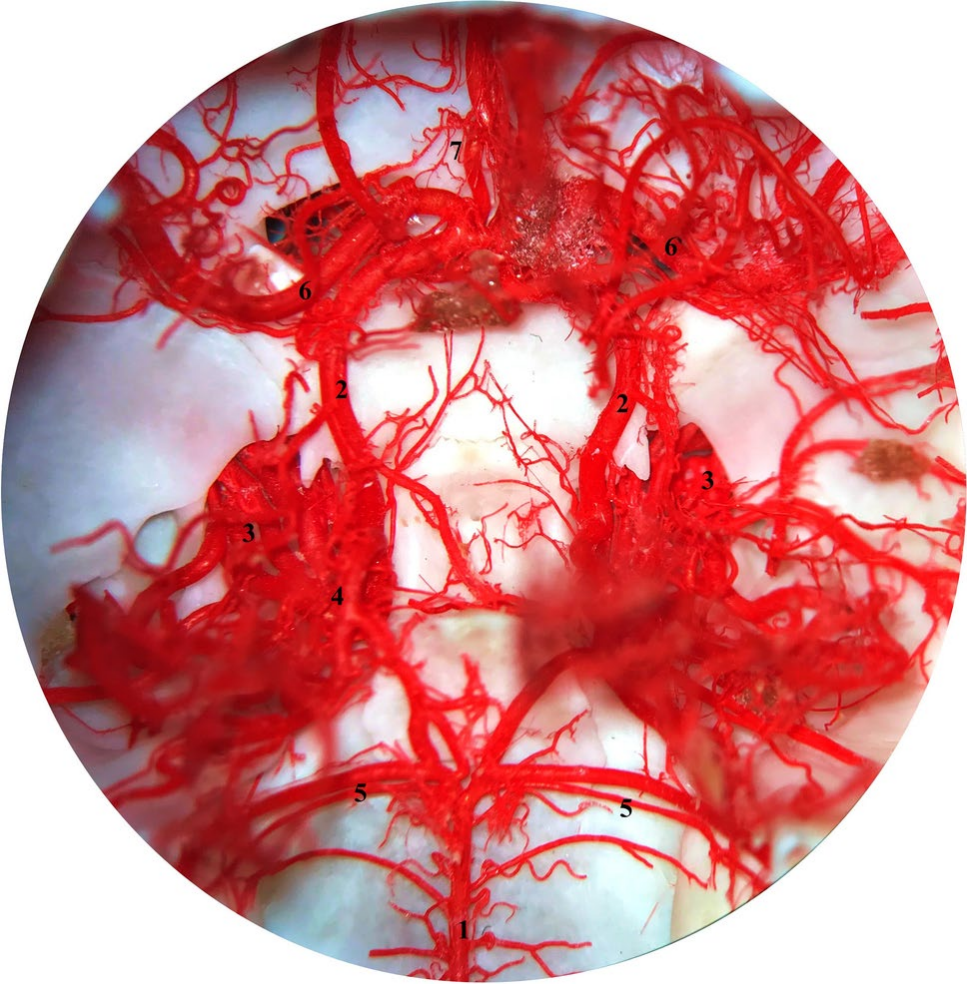
Cerebral arterial circle in a cat (dorsal view). Photography taken by surgical microscope. Corrosion casting method. Mag 10x**.** 1 – basilar artery, 2 – cerebral arterial circle (circle of Willis) main arteries, 3 – rete mirabile of the maxillary artery (retial branches pass through the orbital fissure), 4 – anastomotic branch between rete mirabile and circle of Willis, 5 – rostral cerebellar artery, 6 – middle cerebral artery, 7 – rostral cerebral artery

The internal carotid artery (ICA) is one of the three branches of the common carotid artery, which arises at the level of the thyroid cartilage. The occipital artery and the ascending pharyngeal artery also arise at this level (Kier et al. 2019). The ICA runs dorsally and enters the jugular foramen at the base of the skull without crossing it. The ICA forms an arc of approximately 180° and has the shape of an inverted U. As it continues, it enters the cranial cavity through the carotid canal together with the internal carotid nerve (Getty 1975; Ziemak et al. 2021). The presence of the internal carotid artery is controversial. Some authors claim that cats do not have an extracranial segment of the internal carotid artery (Gillilan 1976; Kiełtyka-Kurc et al. 2015; Kier et al. 2019; Takemura 1982; Ziemak et al. 2021). A recent study that looked at the presence of the internal carotid artery in animals of a different age, confirmed that the ICA is a branch of the CCA. The ICA was present in all foetuses, in 12 of 16 young cats, and was present by 8 weeks of age. On the other hand, the extracranial segment of the ICA was not found in adult animals and in four young cats. Atrophy of the extracranial portion of the ICA occurred in all adult cats, and the carotid sinus was found at the site of ICA origin as a rudiment of the extracranial segment. Interestingly, the carotid sinus was not found in foetuses or younger animals. Obliteration of the ICA occurred in adult and younger cats (Ziemak et al. 2021). The intracranial segment of the ICA forms the cerebral arterial circle with the lateral branch of the weak ascending pharyngeal artery, which is a branch of the CCA and the basilar artery (Fig. 1) (Daniel et al. 1953; Gillilan 1976; Kamijyo and Garcia 1975; Kier et al. 2019; Takemura 1982; Ziemak et al. 2021). In foetuses and kittens, the blood supply to the brain comes from two sources, the ICA and the MA (Ziemak et al. 2021). However, the MA is not direct source for the brain. It forms a specific vascular network which joins to the cerebral arterial circle via anastomotic branch. The cat is not the only member of the feline group in which obliteration of the ICA occurs. ICA obliteration occurs in lions (*Panthera leo*), ocelots (*Leopardus pardalis*), pumas (*Puma concolor*), leopards (*Panthera pardus*), tigers (*Panthera tigris*), and Eurasian lynx (*Lynx lynx*) (Frackowiak and Godynicki 2003; Hsieh and Takemura 1994). Although the canid group is one of the animals in which the ICA is present throughout life, there are certain exceptions in which obliteration of the ICA occurs. Similar arterial patterns of the brain are observed in large Indian civets (*Viverra zibetha*) of the family *Viverridae* and in hyenas (*Hyaena hyaena*) (Bugge 1978; Nickel and Schwarz 1963; Ziemak et al. 2021).

## Other arteries responsible for brain supply

**Fig. 2:**
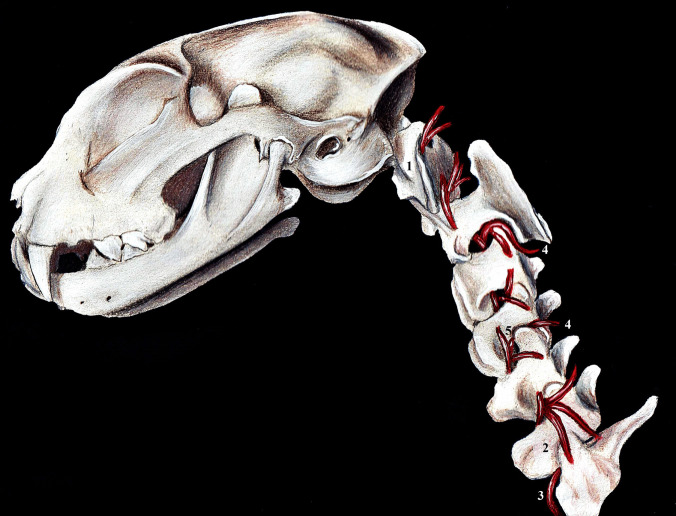
Lateral view on the vertebral arteries that provide the caudal circulation of the brain in the domestic mammals (cat). Illustration was drawn according to original anatomical specimen. 1 – atlas, 2 – sixth cervical vertebra, 3 – vertebral artery entering to the transverse foramen, 4 – dorsal branches of the vertebral artery, 5 – spinal branches of the vertebral artery entering to the intervertebral foramen

**Fig. 3:**
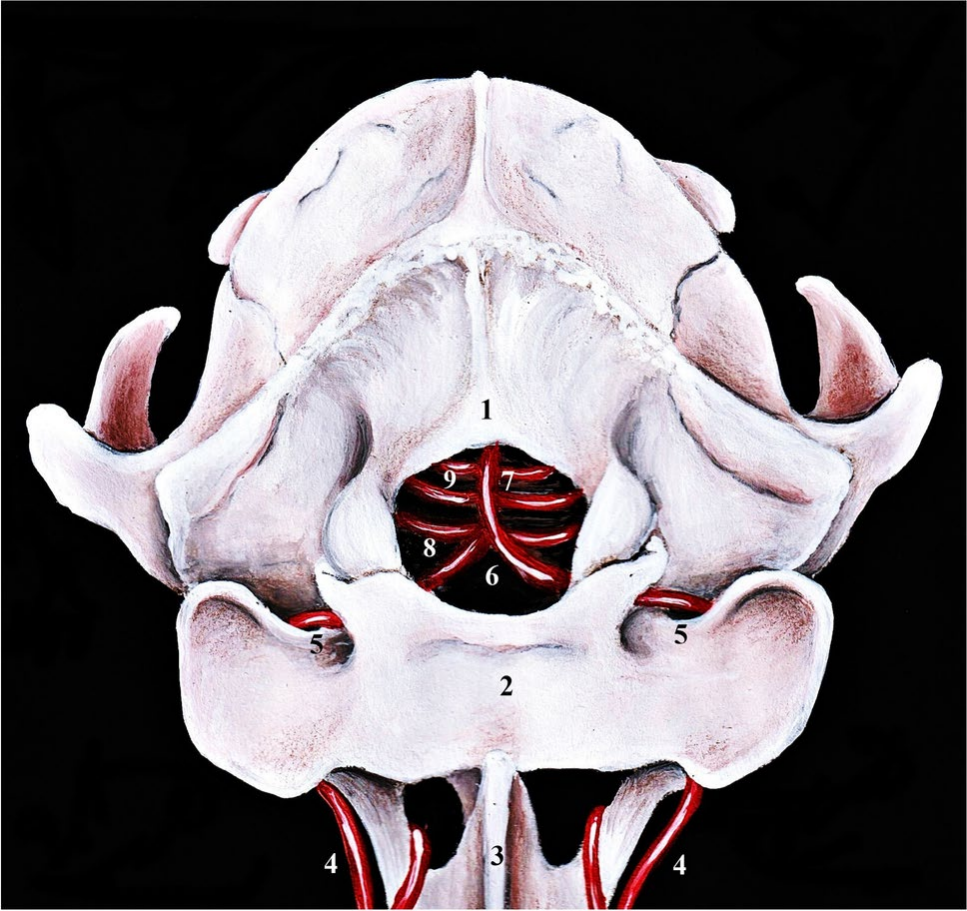
Caudal view on passing of the vertebral arteries to the cranial cavity and formation of the basilar artery in cat. Illustration was drawn according to original anatomical specimen. 1 – occipital bone, 2 – atlas, 3 – axis, 4 – vertebral artery, 5 – vertebral artery passing through alar notch of the atlas, 6 – connection of the both vertebral arteries in the cranial cavity, 7 – basilar artery, 8 – caudal cerebellar artery, 9 – pontine artery

The other branches of the common carotid artery responsible for the arterial supply to the brain are the ascending pharyngeal artery and the occipital artery, which is a branch of the external carotid artery (Kamijyo and Garcia 1975; Kier et al. 2019; Ziemak et al. 2021). The occipital artery has a variable origin. In the cat, it is a branch of the carotid sinus, the CCA, or the ECA. In adult cats, the OA is a branch of the carotid sinus. In contrast to adult cats, the OA in foetuses and young cats arises from the CCA in most cases; in a smaller number of individuals, the OA branches off from the ECA (Ziemak et al. 2021). However, according to Ziemak et al. (2021) and Gillilan (1976), the OA does not represent a direct blood supply to the brain and does not form the basilar artery as it does in dogs. The ascending pharyngeal artery (APA) is considered an alternative supplementary blood source for the brain in cats (Getty 1975). APA was a small branch of the occipital artery in all cats studied. The APA ran on the lateral side of the tympanic bulla and gave off two branches. One branch, located more medially, supplied blood to the pharyngeal wall, and the lateral branch went to the carotid canal. The connection of APA and the ICA was described by Ziemak et al. (2021). The connection between these two arteries is different and has been found in some foetuses, younger cats, and adult cats. The role of APA is to supply blood to the intracranial segment of the ICA. According to the findings, the side branch of APA is responsible for connecting CCA and the intracranial segment of ICA. Similar findings were described by Kamijyo and Garcia (1975) and Kier et al. (2019). Subclavian branches as well as vertebral arteries are also responsible for vascular supply to the brain. The vertebral arteries (VA) run dorso-cranially, emerge from the cranial thoracic aperture and enter the transverse foramen of the sixth cervical vertebra. They then run to the atlas and enter the skull, where they form the basilar artery (Figs. 2 and 3). The basilar artery (BA) is located at the base of the skull, runs rostrally, and enters the cerebral arterial circle on the caudal side of the skull (Fig. 1). The main function of the vertebral arteries is to supply blood (spinal branches) to the cervical portion of the spinal cord (Popesko 1992; Singh 2017).

## Rete mirabile of the maxillary artery in cat

**Fig. 4:**
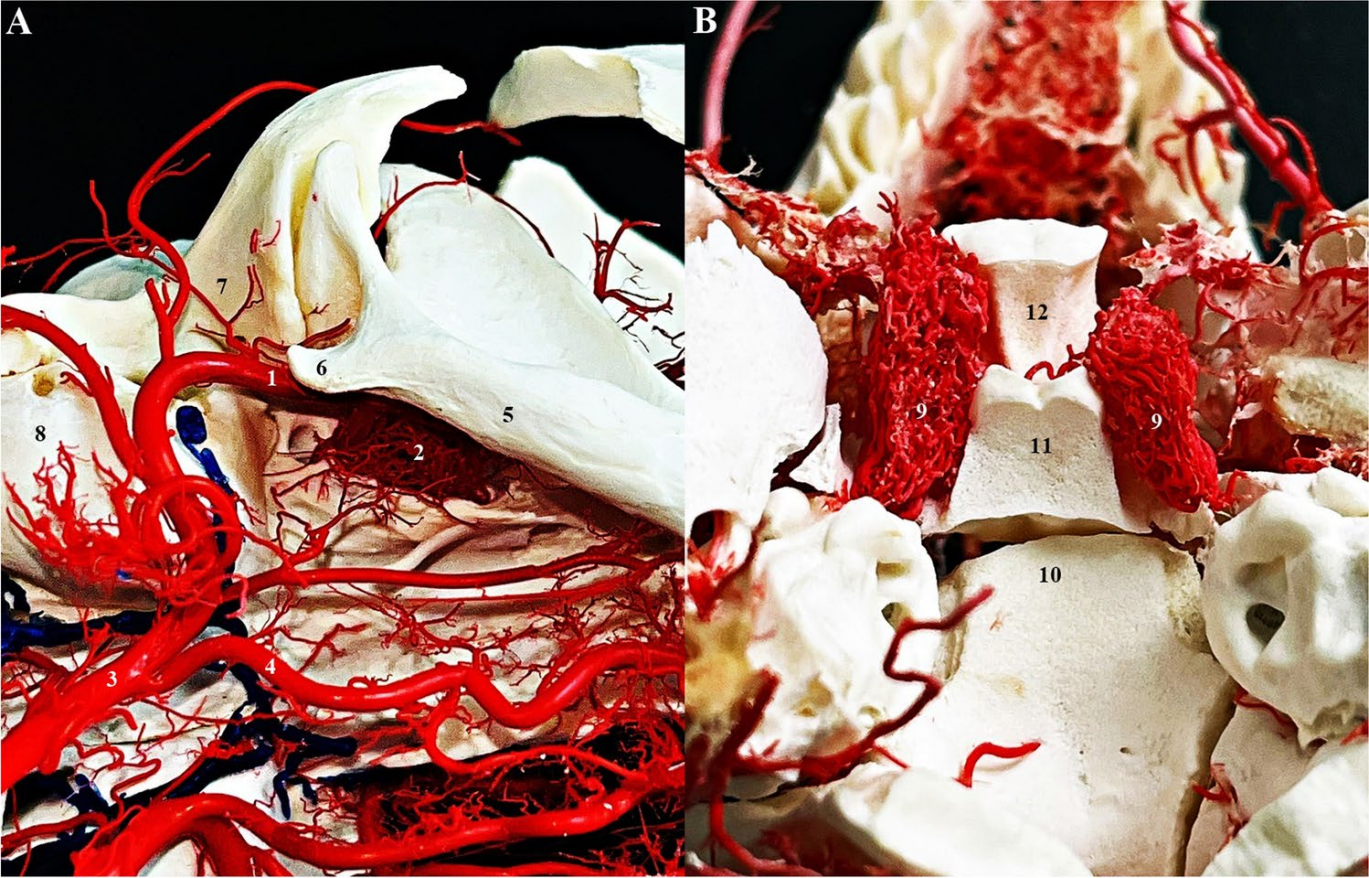
Comparison of topography of the rete mirabile in the cat (**A**) (ventro – lateral view into intermandibular space area) and sheep (**B**) (dorsal view into cranial cavity). Corrosion casting method. 1 – maxillary artery, 2 – rete mirabile, 3 – external carotid artery, 4 – lingual artery, 5 – mandible, 6 – angular process of mandible, 7 – temporal bone, 8 – tympanic bulla, 9 – rostral epidural rete mirabile, 10 – occipital bone, 11 – basisphenoid bone, 12 – presphenoid bone

**Fig. 5:**
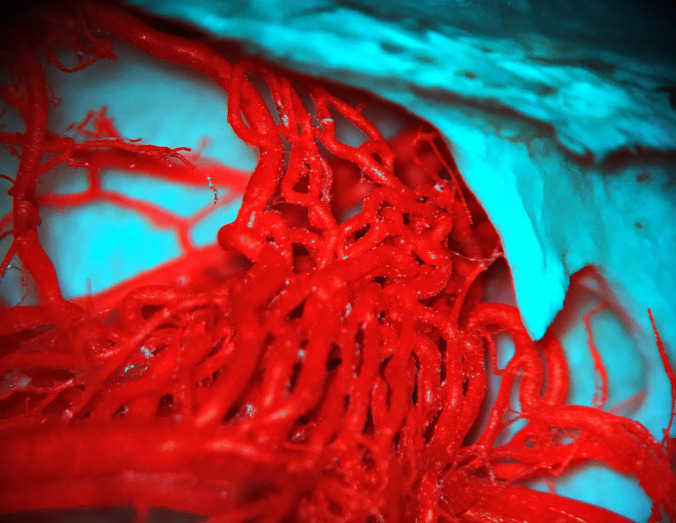
Rete mirabile goes through the orbital fissure into the cranial cavity in cat (lateral view). Photography taken by surgical microscope. Corrosion casting method. Mag 25x. 1 – maxillary artery, 2 – rete mirabile, 3 – retial branches which enter into orbital fissure, 4 – orbital fissure

**Fig. 6:**
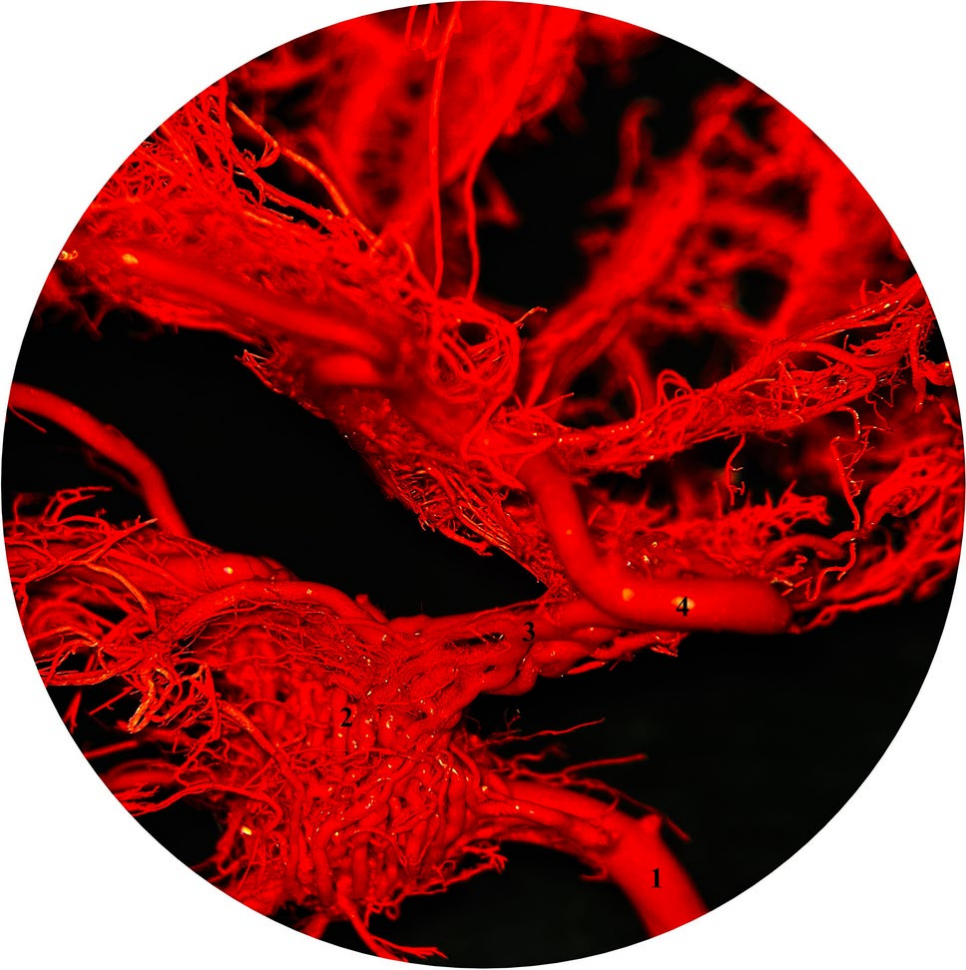
Detailed view on the anastomotic branches from the rete mirabile that connect the maxillary artery and the cerebral arterial circle in cat (medial view). Corrosion casting method. Mag 25x. 1 – maxillary artery, 2 – rete mirabile, 3 – anastomotic branch, 4 – part of the cerebral arterial circle (circle of Willis)

**Fig. 7:**
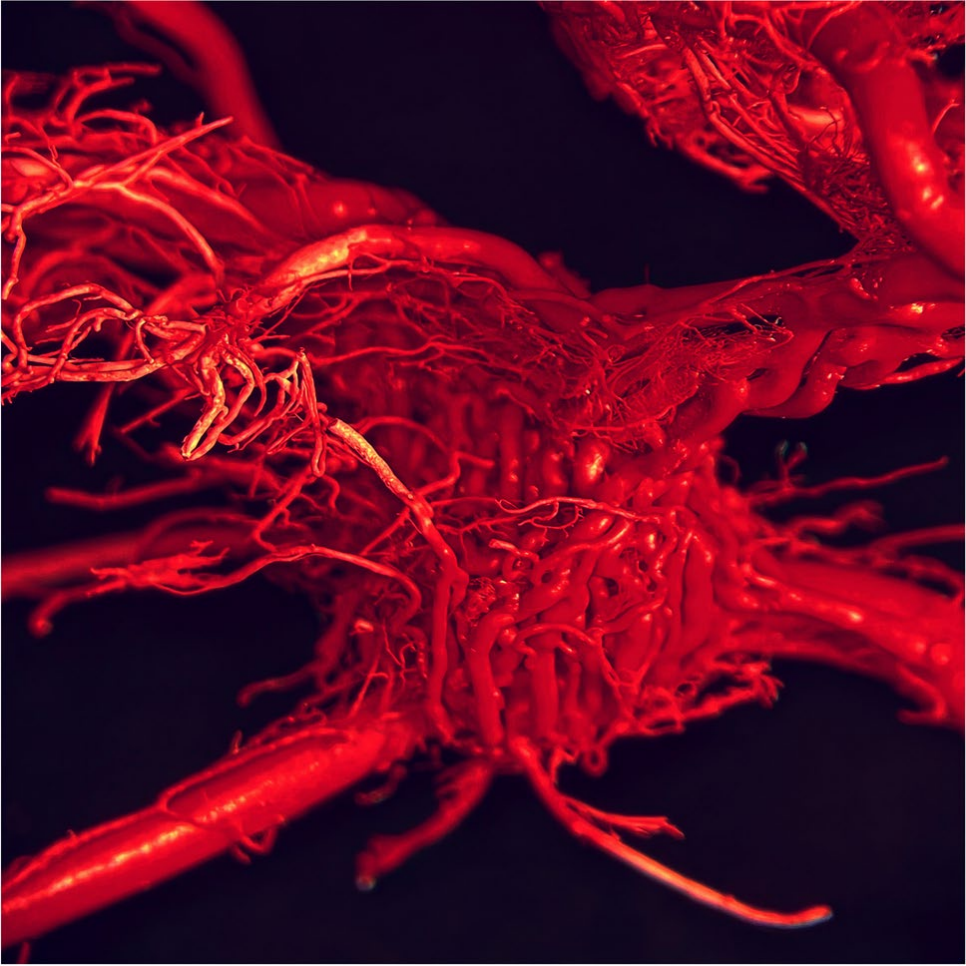
Detailed view on the branches from rete mirabile in cat (medial view). Corrosion casting method. Mag 25x

The rete mirabile (RM) or the wonderful network of the maxillary artery was first described by Tandler in 1899. In the past, various names were used for the rete mirabile, such as "wonderful network" "Wundernetz" "carotid plexus," "carotid rete," and "extracranial rete" (Daniel et al. 1953; Kier et al. 2019; Norris 1906; Takemura 1982). Since then, many anatomical studies have been performed to describe the rete mirabile in cats, with emphasis also on function. The maxillary artery (MA) is a terminal branch of the ECA and provides the main blood supply to the brain in cat. The MA runs rostrally between the ramus of the mandible and the medial pterygoid muscle. There is a close relationship with the maxillary nerve, with MA passing medially under the aforementioned nerve. The pterygoid fossa near the orbital fissure is a place where MA forms a specialized vascular plexus—the rete mirabile. The rete mirabile is a highly specialized vascular plexus that is extracranially localized in the venous sinuses in the cat (Gillilan 1976; Kamijyo and Garcia 1975; Kier et al. 2019; Lombardero et al. 2021; Takemura 1982). In contrast to other domestic mammals, the presence of rete mirabile has been described in pigs and ruminants, but intracranially (Kier et al. 2019) (Fig. 4). The rete mirabile of the maxillary artery is formed by arteries – retial branches with very thin walls that utilize counter current blood flow. The counter current blood flow is effective for heat and gas exchange and ion transport between the blood and other structures (Baker and Hayward 1967; Kier et al. 2019). The rete mirabile extends dorsally and laterally to the apex of the periorbital area and supplies blood to the ophthalmic structures and brain (retial branches). It passes through the orbital fissure into the cavernous sinus in the cranial cavity and joins the cerebral arterial circle (Lombardero et al. 2021; Ziemak et al. 2021) (Fig. 5). The formation of the rete mirabile is not simple, as many studies have described. In the large study by Takemura (1982) of 55 adult cats and 5 new-born kittens, it was described that the rete mirabile of MA consists of the lateral, medial, and anterior retial branches. The lateral branches are approximately 0.47 mm in diameter, numbered 1–6 and are formed by the deep caudal temporal artery and the inferior alveolar artery. The medial branches were formed by the superomedial branch and the inferomedial branch. The superomedial branch was represented by the branch arising from the middle meningeal artery. The inferomedial branch was a direct branch of the MA. The anterior branches were formed by 3 to 7 small arteries derived from the deep rostral temporal artery, buccal artery, and zygomaticus artery. The terminal branches of the rete mirabile of the maxillary artery are described as branches leaving the medial surface of RM. The anastomotic branches – retial branches are well developed and consist of 6 to 9 confluent bundles (posteromedial) which form the anastomotic arteries. These branches are formed from posterosuperior and anteroinferior groups. Both groups unite to form the flattened bundle and enter the orbital fissure. After entering the orbital fissure, these groups enter the cavernous sinus, where the bundle forms a short anastomotic artery that connects to the cerebral arterial circle (Fig. 6). The other terminal arteries were observed in the following order: the external ethmoidal artery, the branches of the extraocular muscles, the meningeal branch, the lacrimal artery, the intraretinal artery, the communicating branch with the external ethmoidal artery, and the temporal branches (Takemura 1982). (Fig. 7).

## Clinical significance of the rete mirabile

**Fig. 8:**
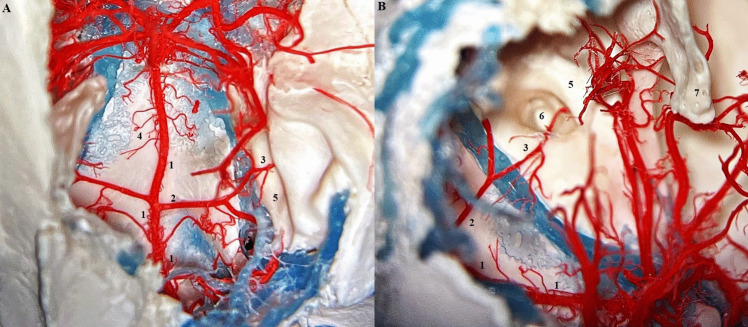
Origin and course of the labyrinthine artery which is branch form the caudal cerebellar artery in this specimen. Dorsal view into the cranial cavity (**A**). Mag. 4x. Oblique view into the cranial cavity with focused to entrance into the internal acoustic meatus of the temporal bone (**B**). Mag 10x. Corrosion casting method. 1 – basilar artery, 2 – caudal cerebellar artery, 3 – labyrinthine artery, 4 – pontine arteries, 5 – petrous part of the temporal bone, 6 – internal acoustic meatus, 7 – cerebellar tentorium

Cats are one of the most common companion animals, and they differ from dogs in several important ways. Given the centrality of anatomy in high-quality medicine, the treatment of the feline mandible has many important features, most of which are due to the uniqueness of its anatomy compared with that of the dog. Knowledge of the functional anatomy of the feline mandible provides us with clues for appropriate treatment of the feline patient and minimises the incidence of potential iatrogenic injuries (Lombardero et al. 2021). The role of the maxillary artery and rete mirabile has been discussed in detail in recent publications focusing on their various physiological functions (Lombardero et al. 2021; O’Brien and Bourke 2015; O’Brien 2018, 2020; Strauss et al. 2017). Cats recovering from general anaesthesia have been reported to develop transient or permanent blindness, alone or with central neurologic deficits. The blindness was initially attributed to anaesthesia-induced hypoxia and hypotension. However more recently, the use of mouth gags and prolonged maximal mouth opening have been implicated as factors in the development of this complication. The mouth gag is a commonly used tool in veterinary medicine for oral and trans – oral procedures in cats. The maxillary arteries of cats are at risk for vascular occlusion during maximum mouth opening. In cats and dogs, the maxillary artery passes through the caudal portion of the mandible, but only cats are at increased risk of cerebral ischemia when the mouth is wide open because the mandibular angular process presses against the area through which the maxillary artery passes. This reduces maxillary artery blood flow to some degree. Crucial to this phenomenon, however, is another feature: the internal carotid artery (which conducts the main blood flow to the brain, retina, and inner ear) is functionally absent in cats, so the majority of blood going to the brain is supplied by the bilateral maxillary arteries. The longer the pressure persists, the higher the risk of occurrence of brain and retinal ischemia and/or blindness and/or deafness (Barton-Lamb et al. 2013, Lombardero et al. 2021; Reiter and Soltero-Rivera 2014; Scrivani et al. 2014). A reduction in maxillary artery blood flow due to mediolateral compression of the maxillary artery between the angular process of the mandible and the tympanic bulla has been reported in cats when the mouth is open during intraoral procedures (Scrivani et al. 2014). Based on a few studies, deafness is caused by decreased blood flow to the inner ear when procedures require prolonged opening of the mouth (Barton-Lamb et al. 2013; Stevens-Sparks and Strain 2010). The inner ear in all living beings is very vulnerable to ischemic changes because of lack of collateral blood supply (Glueckert et al. 2018). The labyrinthine artery is the only artery responsible for blood supply of the inner ear in cats. However, sufficient description of the blood supply of the inner ear (cochlea) is questionable. Stevens-Sparks and Strain (2010) noted that the labyrinthine artery derives from the vertebral artery via the middle cerebellar artery and the basilar artery in dogs (Anderson and Anderson 1994). Getty (1975) observed the labyrinthine artery, which originated from the caudal cerebellar artery in cats. Nomina Anatomica Veterinaria (NAV) recognizes that the labyrinthine artery is a branch of the basilar artery in carnivores (WAVA 2017). Currently, according to our reviews, there is only one detailed study that addresses the anatomy of the labyrinthine artery in cats. The labyrinthine arteries were not branches from the basilar artery in any case (Bernstein and Silverstein 1966). Branches (anterior branch) from the anterior cerebellar artery went into the internal acoustic meatus. The labyrinthine arteries are observed in the internal acoustic meatus between the facial nerve and the vestibulocochlear nerve. In a few cases, authors observed a single labyrinthine artery which arose from the anterior branch of the anterior cerebellar artery. An in vivo study that deals with obliteration of the brain base arteries gave interesting results. This study found that obliteration of the anterior cerebellar artery and the labyrinthine artery may produce negative changes in the inner ear (Bernstein and Silverstein 1966). Term anterior branch and the anterior cerebellar artery is not correct in the veterinary anatomy. Such an artery does not exist in animals. Instead, there is a rostral branch of the rostral cerebellar artery. The term rostral cerebellar artery is also in accordance with NAV 2017. The evidence leads us to believe that the labyrinthine artery is the branch from the caudal cerebellar artery and it is possible there are individual variations in the origin of this artery (Fig. 8). Like Getty (1975), we recognize that the labyrinthine artery is a branch from the caudal cerebellar artery, as we could observe on our specimens. It is also necessary to note that in this book the blood supply of the brain in a cat is very precisely described (Getty 1975). However, the mechanism of the mouth opening and association with topography of the vertebral arteries and the basilar artery is not elucidated. There must be another hypoperfusion mechanism of the labyrinthine arteries. There might be individual vascular variation from the origin of the labyrinthine artery (Stevens-Sparks and Strain 2010). Hypoperfusion in the labyrinthine artery causes functional disturbances and shortage of nutrients, oxygen, and accumulation of toxic substances in the inner ear (Nuttall 1987). Barton-Lamb et al. (2013) described that brain cortex injury can be present in cats with inner ear dysfunction and deafness, but in the mentioned study the authors did not find any cat with brain cortex injury based on dynamic computed tomography during dental interventions with maximal mouth opening. This may be due to other alternative routes of blood supply to the brain, such as the basilar artery, which compensates the blood flow. Redirection of the blood flow via the cerebral arterial circle is caused by occlusion of the maxillary artery (King 1987). According to our hypothesis, redirection of blood flow can cause hemodynamic phenomenon in the cerebral arterial circle and leads to other blood flow disorders in different arteries. Barton-Lamb et al. (2013) described that the BAER (Brainstem Auditory Evoked Response) examination in a cat confirmed depression of signals during mouth opening. It is recommended to reduce the degree of mouth opening or close the mouth every few minutes during oral examination or surgery to preserve the blood flow (De Miguel et al. 2013). Cerebrovascular accidents are described in dogs and cats, but the incidence is rarely reported in cats (Altay et al. 2011; Whittaker et al. 2018). Because of the difference in the vascular system of the brain between the two species, cats may have a lower rate of cerebrovascular incidents. The reasons and mechanisms of protection are not yet clear (Whittaker et al. 2018). Some mammals are able to lower their hypothalamic temperature below the temperature of the carotid arterial blood, a process called selective brain cooling. The necessary anatomical structure that enables this physiological process, the rete mirabile, is present and particularly well developed in antelope, cattle, sheep, and goats. First described in the domestic cat, the seemingly obvious function attributed to selective brain cooling was to protect the brain from thermal damage. However, hyperthermia is not a prerequisite for selective brain cooling, and selective brain cooling can occur at any time of day, even when the arterial blood temperature in the carotid artery is relatively low. Selective brain cooling has been shown to function primarily as a water conservation mechanism, allowing artiodactyls to conserve more than half of their daily water requirements (Strauss et al. 2017).

## Conclusion

The blood supply to the brain is critical for the survival of individuals. Its damage can lead to serious or even fatal consequences. The maxillary artery forms a specific network in the pterygoid fossa called the rete mirabile, which consists of retial branches that pass through the orbital fissure into the cranial cavity. The maxillary arteries of cats are at risk for vascular occlusion during maximum mouth opening as it passes through the caudal portion of the mandible leading cats to have an increased risk of cerebral ischemia when the mouth is wide open. Crucial to this phenomenon, however, is another feature: the internal carotid artery (which conducts the main blood flow to the brain, retina, and inner ear) is functionally absent in cats, so the majority of blood flow to the brain is supplied by the bilateral maxillary arteries with its rete mirabile. The longer the pressure persists, the higher the risk of occurrence of brain and retinal ischemia and/or blindness and/or deafness. Precise knowledge of these anatomical structures are vital in order to be able to perform highly safe procedures, surgeries, and to minimize the risk of iatrogenic diseases in animals.

## Data Availability

The data are published in manuscript.
